# Optical Force Effects of Rayleigh Particles by Cylindrical Vector Beams

**DOI:** 10.3390/nano14080691

**Published:** 2024-04-17

**Authors:** Yuting Zhao, Liqiang Zhou, Xiaotong Jiang, Linwei Zhu, Qiang Shi

**Affiliations:** 1 School of Physics and Optoelectronic Engineering, Ludong University, Yantai 264025, China; zhaoyuting809@gmail.com (Y.Z.);; 2 Moji-Nano Technology Co., Ltd., Yantai 264006, China

**Keywords:** cylindrical-vector beams, optical force, polarization, Rayleigh particles

## Abstract

High-order cylindrical vector beams possess flexible spatial polarization and exhibit new effects and phenomena that can expand the functionality and enhance the capability of optical systems. However, building a general analytical model for highly focused beams with different polarization orders remains a challenge. Here, we elaborately develop the vector theory of high-order cylindrical vector beams in a high numerical aperture focusing system and achieve the vectorial diffraction integrals for describing the tight focusing field with the space-variant distribution of polarization orders within the framework of Richards–Wolf diffraction theory. The analytical formulae include the exact three Cartesian components of electric and magnetic distributions in the tightly focused region. Additionally, utilizing the analytical formulae, we can achieve the gradient force, scattering force, and curl-spin force exerted on Rayleigh particles trapped by high-order cylindrical vector beams. These results are crucial for improving the design and engineering of the tightly focused field by modulating the polarization orders of high-order cylindrical vector beams, particularly for applications such as optical tweezers and optical manipulation. This theoretical analysis also extends to the calculation of complicated optical vortex vector fields and the design of diffractive optical elements with high diffraction efficiency and resolution.

## 1. Introduction

In recent years, there has been an extensive exploration of cylindrical vector (CV) beams due to their spatially variant polarization orders [1,2,3,4,5,6]. Researchers have developed numerous systems for generating CV beams with exotic properties [7,8,9,10,11,12] and have applied them in various applications, such as optical manipulation [13,14,15,16,17,18,19], the spin-orbit hall effect [20,21,22], multiplex communication [23], and super-resolution imaging [24]. These applications have sparked growing interest in the high numerical aperture (NA) focusing on the characteristics of CV beams and their properties in optical capture and manipulation. CV beams are vector-beam solutions of Maxwell’s equations that exhibit axial symmetry in both amplitude and phase. Furthermore, the additional degree of freedom in high-order CV beam allows for engineering the characteristics of the focal field to tailor the distribution of the optical force, thereby addressing specific requirements of optical tweezer systems. In 2004, Zhan demonstrated that metallic Rayleigh particles with strong scattering and absorption forces could be trapped using tightly focused radial polarization beams [25]. In 2017, Li et al. integrated high-order CV beams with variable polarization topological charge to analyze the spin momentum and torque exerted on the trapped particles [26]. Moreover, forces partially associated with the spin of the internal energy flow can induce particle rotation and influence their motion [27,28]. The aforementioned examples underscore the significance of studying vector diffraction theory near the focus of high-order CV beams.

Thus, the different analytical models of vector formalism in tightly focused fields have been proposed further to explore the focusing characteristics of highly focused CV beams. To achieve high diffraction efficiency and high resolution of diffractive optical elements, computational approaches involving various vector models have been utilized in the vicinity of the focus [29,30]. Rui et al. presented a novel strategy to manipulate the captured metallic nanoparticles by using an engineered azimuthally polarized optical field and gave the complete electric field expression to predict the motion behavior of the trapped nanoparticles [31]. However, it does not include the case of higher-order polarization. Additionally, Chen et al. proved that an optical pulling force could pull a particle toward the source via a backward scattering force [32], but the complete analytical models for the fast calculation were not provided. In summary, there is still a lack of an analytical model based on rigorous diffraction theory to further investigate the multi-dimensional force aroused by the CV beams, including arbitrary polarization orders.

In this paper, we elaborately calculate the analytical formulae of electric and magnetic field distributions based on the Richards–Wolf vector diffraction integral theory [33]. In addition, the gradient force, scattering force, and curl-spin force induced by the high-order CV beams are calculated in detail. Further, we find that high-order polarization vector beams can produce pulling force along the axis, which helps understand the propagation behavior of scattering force in tightly focused high-order beams. The universality of the expressions in arbitrary polarization order CV beams simplifies multi-parameter calculation in the tightly focused region.

## 2. Theoretical Model

### 2.1. Calculation of Cylindrical Vector Beam with Arbitrary Polarization Distribution

**Figure 1:**
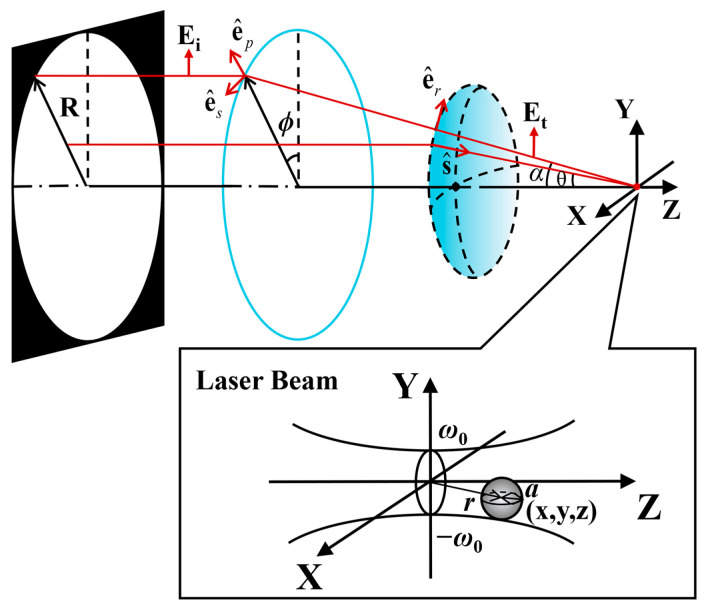
Schematic diagram of focusing system geometry.

As depicted in Figure 1, we assume that an exemplar paraxial CV beam passes through an aperture with radius *R* and is focused by a Fourier lens. A Rayleigh particle with radius *a* is captured near the focal region, where *a* is significantly smaller than the wavelength of the beam.

The paraxial cylindrical vector beam propagating in the direction of the z axis has a transverse electric field in the x–y plane perpendicular to the direction of propagation, which can be expressed as follows:

(1) $$E_{i}=\cos(m\phi +\phi_{0})e_{x}+\sin(m\phi +\phi_{0})e_{y},$$

where *m* is the polarization order, *ϕ* is the azimuthal angle, and the initial phase *ϕ*_0_ is constant. The **e_x_** and **e_y_** are the unit vectors in the x and y directions.

**Figure 2:**
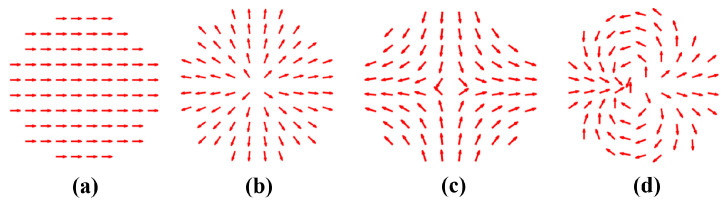
Polarization distributions of incident vector beam with different orders: (**a**) *m* = 0, *ϕ*_0_ = 0, (**b**) *m* = 1, *ϕ*_0_ = 0, (**c**) *m* = −1, *ϕ*_0_ = 0, and (**d**) *m* = 2, *ϕ*_0_ = 0. Arrows indicate the direction of polarization at the arrow location.

Any polarization order of the beam in a transverse field can be decomposed into two basic polarizations: radial and azimuthal. Due to the polarization singularity at the center, different CV beams exhibit a doughnut-shaped intensity distribution [34]. The weight of the radial and azimuth polarization components varies along the azimuthal angle. Figure 2 shows the polarization distribution of the CV beams with *m* = 0, 1, −1, 2, and *ϕ*_0_ = 0. It demonstrates that the polarization structure is determined by the polarization orders, which dictate the spatial distribution and weight of radial and azimuthal polarization components. Thus, the different types of CV beams can be simulated by adjusting the polarization order *m*. In the transmission field,

(2) $$e_{p}=\left(\begin{array}{c} \cos\phi \\ \sin\phi \\ 0 \end{array}\right),e_{s}=\left(\begin{array}{c} -\sin\phi \\ \cos\phi \\ 0 \end{array}\right),e_{r}=\left(\begin{array}{c} \cos\phi \cos\theta \\ \sin\phi \cos\theta \\ -\sin\theta \end{array}\right),$$

where **e_p_**, **e_s_**, and **e_r_** are the unit vectors as shown in Figure 1, and *θ* is the deflection angle. Then, the transmission field can be expressed as follows:

(3) $$E_{t}(\theta ,\phi )=t_{p}(E_{i}\cdot e_{p})\cdot e_{r}+t_{s}(E_{i}\cdot e_{s})\cdot e_{s},$$

by substituting from Equations (1) and (2) into Equation (3), we can obtain the following:

(4) $$E_{t}=\left(\begin{array}{c} -\sin\delta \sin\phi +\cos\delta \cos\theta \cos\phi \\ \sin\delta \cos\phi +\cos\delta \cos\theta \cos\phi \\ -\cos\delta \sin\theta \end{array}\right).$$

Here, *δ = (m* − 1*) ϕ* + *ϕ*_0_. For a high NA focusing system, the electric field vector in the focal area can be obtained by the diffraction integral of **a**. Here, **a** represents the ‘strength factors’ of the electric field, and the relation between **a** and the electric field in image space is as follows [33]:

(5) $$a=fl\sqrt{\cos\theta }E_{t},$$

where *f* is the focal length, and *l* is the amplitude factor that depends on the power. On substituting Equation (4) into Equation (5), we obtain the following:

(6) $$\left\{\begin{array}{l} a_{x}=fl\sqrt{\cos\theta }(-\sin\delta \sin\phi +\cos\delta \cos\theta \cos\phi ),\\ a_{y}=fl\sqrt{\cos\theta }(\sin\delta \cos\phi +\cos\delta \cos\theta \cos\phi ),\\ a_{z}=fl\sqrt{\cos\theta }\left(-\cos\delta \sin\theta \right). \end{array}\right.$$

The ‘strength factors’ **b** of the magnetic field is related by the formula:

(7) b=κ(s×a).

Here, *κ* = 1/i*ωμ*_0_. *ω* is the angular frequency of the optical field, and *μ*_0_ is the permeability in vacuum. **s** is the unit vector along the ray in the image space. Its three components can be expressed as **s** = (sin*θ*cos*φ*, sin*θ*sin*φ*, cos*θ*). Substituting Equation (6) into Equation (7), we obtain the Cartesian components of the strength vectors **b**:

(8) $$\left\{\begin{array}{l} b_{x}=\kappa fl\sqrt{\cos\theta }(-\cos\delta \sin\phi -\sin\delta \cos\theta \cos\phi ),\\ b_{y}=\kappa fl\sqrt{\cos\theta }(\cos\delta \cos\phi -\sin\delta \cos\theta \cos\phi ),\\ b_{z}=\kappa fl\sqrt{\cos\theta }(\sin\delta \sin\theta ). \end{array}\right.$$

According to the Richards–Wolf vector diffraction integral theory [33], the electric and magnetic fields for an aberration elimination system at any point near the focal point can be written as follows:

(9) $$\left\{\begin{array}{c} E\left(x,y,z\right)=\frac{1}{i\lambda }\iint_{\Omega }\frac{a\left(s_{x},s_{y}\right)}{s_{z}}\exp(ik\left[s_{x}x+s_{y}y+s_{z}z\right])ds_{x}ds_{y},\\ H(x,y,z)=\frac{1}{i\lambda }\iint_{\Omega }\frac{b(s_{x},s_{y})}{s_{z}}\exp(ik\left[s_{x}x+s_{y}y+s_{z}z\right])ds_{x}ds_{y}, \end{array}\right.$$

where k = 2π/λ is the wave number, and λ is the wavelength in the vacuum.

To describe the electric and magnetic fields in detail, the quantity ds_x_ds_y_/s_z_, which enters the basic diffraction integral, represents the element dΩ of the solid angle and is given by ds_x_ds_y_/s_z_ = dΩ = sin*θ*d*θ*d*φ*. Substituting from Equation (6) into Equation (9), we obtain the following expressions for the Cartesian components *E_x_*, *E_y_*, and *E_z_*:

(10) $$\left\{\begin{array}{l} E_{x}=\int_{0}^{2\pi }\int_{0}^{\alpha }u(-\sin\delta \sin\phi +\cos\delta \cos\theta \cos\phi )d\phi d\theta ,\\ E_{y}=\int_{0}^{2\pi }\int_{0}^{\alpha }u(\sin\delta \cos\phi +\cos\delta \cos\theta \sin\phi )d\phi d\theta ,\;\\ E_{z}=\int_{0}^{2\pi }\int_{0}^{\alpha }u(-\cos\delta \sin\theta )d\phi d\theta , \end{array}\right.$$

where *α* is the maximum allowed incident angle determined by the numerical aperture of the objective lens, and *u* = −*i*π*fl/(*π*λ*)sin*θ*cos^1/2^*θ*exp{*ik*[*r*sin*θ*cos(*ϕ − φ*) + zcos*θ*]}. In the same way, on substituting from Equation (8) into Equation (9), we obtain the following expressions of magnetic fields for the Cartesian components *H*_x_, *H*_y_, and *H*_z_:

(11) $$\left\{\begin{array}{l} H_{x}=\kappa \int_{0}^{2\pi }\int_{0}^{\alpha }u(-\cos\delta \sin\phi -\sin\delta \cos\theta \cos\phi )d\phi d\theta ,\\ H_{y}=\kappa \int_{0}^{2\pi }\int_{0}^{\alpha }u(\cos\delta \cos\phi -\sin\delta \cos\theta \cos\phi )d\phi d\theta ,\\ H_{z}=\kappa \int_{0}^{2\pi }\int_{0}^{\alpha }u(\sin\delta \sin\theta )d\phi d\theta . \end{array}\right.$$

The integration concerning *ϕ* can immediately be carried out with the help of the following formulae, which are valid for any integral value of *n* [35]:

(12) $$\int_{0}^{2\pi }\exp(in\phi )\exp[ikr\sin\theta \cos(\phi -\varphi )]d\phi =2\pi i^{n}J_{n}(kr\sin\theta )\exp(in\varphi ).$$

Here, *J*_n_ is the Bessel function of the first kind with order *n*.

From Equations (10)–(12), we finally obtain the following expressions for the components of the electric field vectors in the focused field:

(13) $$\left\{\begin{array}{l} E_{x}=i^{m-1}e^{i\phi_{0}}(I_{0}+I_{1})-i^{1-m}e^{-i\phi_{0}}(I_{2}+I_{3}),\\ E_{y}=i^{m}e^{i\phi_{0}}(I_{1}-I_{0})-i^{-m}e^{-i\phi_{0}}(I_{3}-I_{2}),\\ E_{z}=i^{m}e^{i\phi_{0}}I_{4}-i^{-m}e^{-i\phi_{0}}I_{5}, \end{array}\right.$$

(14) $$\left\{\begin{array}{l} H_{x}=\kappa [i^{m}e^{-i\phi_{0}}(I_{0}-I_{1})+i^{-m}e^{i\phi_{0}}(I_{2}-I_{3})],\\ H_{y}=\kappa [i^{m-1}e^{-i\phi_{0}}(I_{0}+I_{1})+i^{1-m}e^{i\phi_{0}}(I_{2}-I_{3})],\\ H_{z}=\kappa [-i^{m-1}e^{-i\phi_{0}}I_{4}+i^{1-m}e^{i\phi_{0}}I_{5}], \end{array}\right.$$

where

(15) $$\left\{\begin{array}{l} I_{0}=\int_{0}^{\alpha }u_{1}(1+\cos\theta )J_{m}(kr\sin\theta )d\theta ,\\ I_{1}=e^{(m-2)i\varphi }\int_{0}^{\alpha }u_{1}(1-\cos\theta )J_{m-2}(kr\sin\theta )d\theta ,\\ I_{2}=e^{(2-m)i\varphi }\int_{0}^{\alpha }u_{1}(1-\cos\theta )J_{2-m}(kr\sin\theta )d\theta ,\\ I_{3}=e^{-im\varphi }\int_{0}^{\alpha }u_{1}(1+\cos\theta )J_{-m}(kr\sin\theta )d\theta ,\\ I_{4}=2e^{(m-1)i\varphi }\int_{0}^{\alpha }u_{1}\sin\theta J_{m-1}(kr\sin\theta )d\theta ,\\ I_{5}=2e^{(1-m)i\varphi }\int_{0}^{\alpha }u_{1}\sin\theta J_{1-m}(kr\sin\theta )d\theta , \end{array}\right.$$

with *u*_1_ = π *fl*/(2*λ*) sin*θ*cos^1/2^*θ*exp(*ikz*cos*θ*). Hence, we can calculate the distribution of the electromagnetic field of a high-order CV beam with arbitrary polarization orders in the tight focusing system. Therefore, the distribution of optical forces can be obtained. We will discuss these characteristics in the following sections.

### 2.2. Theory of Optical Force on a Rayleigh Particle

It is assumed that a Rayleigh particle with a radius smaller than the wavelength is near the focusing region, and its motion is controlled by the force of light [36]. Furthermore, in non-magnetoelectric dipolar particles, there are no magnetic dipoles [37]. The particle can be considered simply as a dipole in the calculation [38,39]. The expression of the time-averaged optical force can be written as follows [40,41,42]:

(16) ⟨F⟩=14Re⁡{α1}∇|E|2+σcS+σc∇×LS.

The first part of Equation (16) is the gradient force, which depends on the strength of the electric field, and it can be written as follows:

(17) Fg=14Re⁡α1∇E2.

Here, *α*_1_ is the polarizability given by the following:

(18) $$\alpha_{1}=\frac{\alpha_{0}}{1-i\alpha_{0}k^{3}/(6\pi \varepsilon_{0})},$$

with *α*_0_ = 4π*ε*_0_*a*^3^[(*ε* − 1)/(*ε* + 2)], and *ε* is the relative permittivity of the nanoparticle, and *a* is the radius of the nanoparticle.

The second part in Equation (16) is the scattering force, and it is proportional to the time-averaged Poynting vector:

(19) $$F_{s}=\frac{\sigma }{c}\left\langle S\right\rangle ,$$

where S=12Re⁡{E×H*} is the time-averaged Poynting vector. *σ* = *k*Im{α_1_}/*ε*_0_ is the total cross-section of the particle. *c* is the speed of light in vacuum.

The third part of the right hand in Equation (16) is the curl-spin force, which is associated with the spin density of the light field. The expression is as follows:

(20) $$F_{c}=\sigma \left\{c\nabla \times \left\langle L_{S}\right\rangle \right\},$$

where $\left\langle L_{S}\right\rangle =\frac{\varepsilon 0}{4\omega i}\{E\times E^{*}\}$ is the time-averaged spin density of a transverse electromagnetic field [40]. *ω* refers to the angular frequency of the time-harmonic. For low numerical aperture illumination, this term is usually ignored, and for plane wave illumination with linear polarization, this term is zero.

In summary, light forces on Rayleigh particles can be described as the sum of three terms: the gradient force, the radiation pressure force, and the curl-spin force. We will discuss these forces in detail by employing the derived formulae and the variation in these forces in vector beams with different polarization orders; then, the trapping behavior of Rayleigh particles in tightly focused systems can be fully verified theoretically.

## 3. Results and Discussion

### 3.1. Electric and Magnetic Fields of High-Order Vector Beams

**Figure 3:**
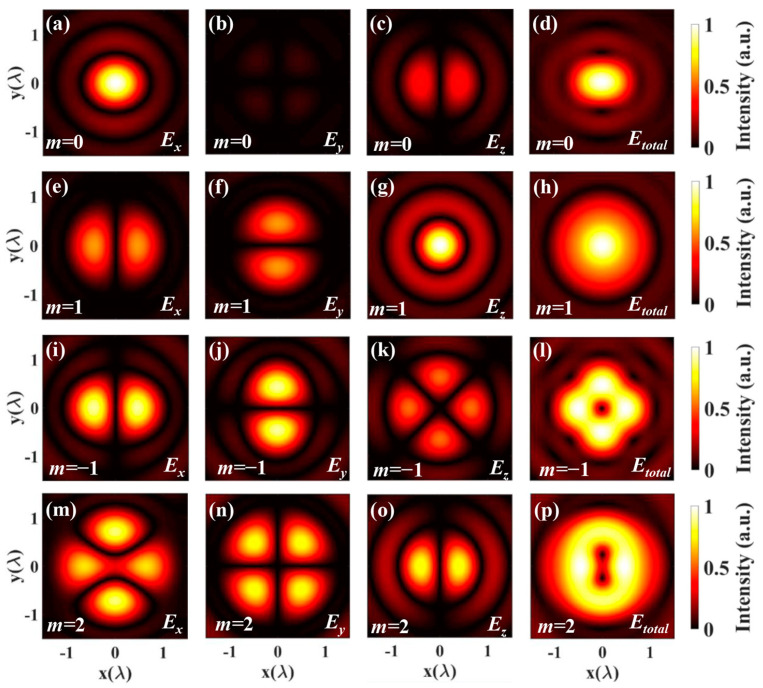
The intensity distribution on the focal plane of vector beams *E_x_*, *E_y_*, *E_z_* and *E_total_* with different orders: (**a**–**d**) *m* = 0, (**e**–**h**) *m* = 1, (**i**–**l**) *m* = −1, (**m**–**p**) *m* = 2.

Now, we investigate the electromagnetic intensity distribution of the light field in tightly focused systems. We use the derived integral of the electric field to simulate the electric field intensity distribution, as shown in Figure 3. It shows the Cartesian coordinate components and total electric field intensity distribution of CV beams with polarization orders of 0, 1, −1, and 2 in the focusing field. The simulations are for the objective with NA = 0.9, and the initial phase *ϕ*_0_ = 0. The objective filling factor was assumed to be one.

As shown in Figure 3, the electric field presents the different distributions of the different polarization orders. *E_x_*, *E_y_*, and *E_z_* represent the x-component, the y-component, the z-component, respectively. *E_total_ = (E_x_*^2^ *+ E_y_*^2^ *+ E_z_*^2^*)*^1/2^ represents the total of the intensity patterns. In the case of the polarization order *m* = 0 (shown in the top row), the electric field intensity distribution is the same as linear polarization [43]. When the polarization order *m* = 1 in the second row, the electric field intensity distribution is the same as radial polarization. Furthermore, it is clear to see that several petals are arranged symmetrically on the ring when *m* = −1 in the third row. Theoretically, the petal-like patterns with the number of 2(|*m*| + 1) will appear for *m* < 0. And when *m* > 1, the total intensity with the arrangement of hot spots with the number of 2(*m* − 1) in the bottom row. This kind of intensity structure has a potential application value in multiple-particle trapping.

**Figure 4:**
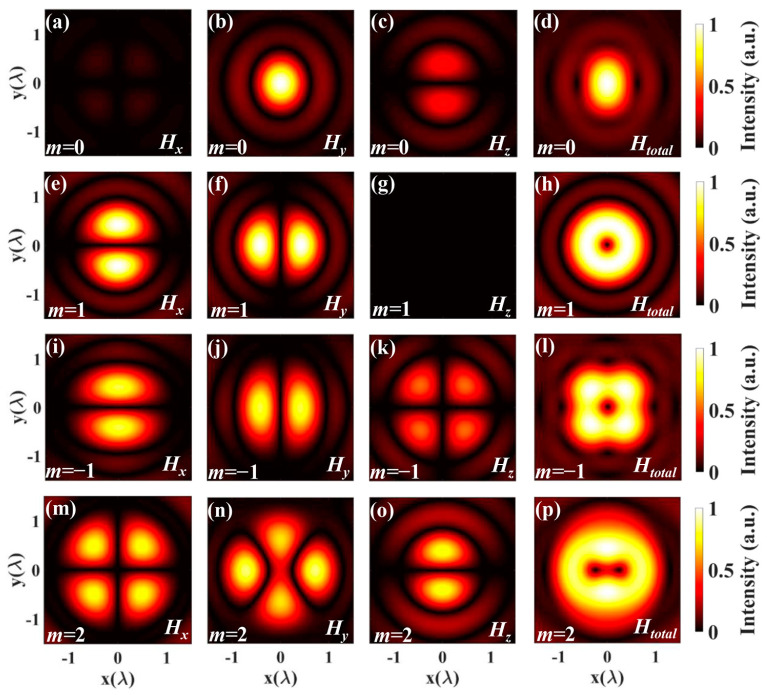
The intensity distribution on the focal plane of vector beams *H_x_*, *H_y_*, *H_z_* and *H_total_* with different orders: (**a**–**d**) *m* = 0, (**e**–**h**) *m* = 1, (**i**–**l**) *m* = −1, (**m**–**p**) *m* = 2.

Figure 4 shows *H_x_*, *H_y_*, and *H_z_* that represent the x-component, the y-component, and the z-component, respectively. *H_total_* = (*H_x_*^2^ + *H_y_*^2^ + *H_z_*^2^)^1/2^ represents the total magnetic field of the high-order vector beams. Like the electric field intensity distribution, it is observed that several hot spots are arranged symmetrically for *m* < 0 or *m* > 1 on the ring. By comparing Figure 3 and Figure 4, it is demonstrated that the electric and magnetic components are perpendicular to each other. Differently, when *m* = 1, the distribution of electric field intensity shows the solid core, while the distribution of magnetic field intensity has a hollow center.

**Figure 5:**
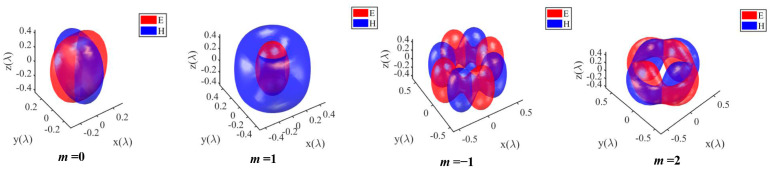
The iso-surface intensity distribution of the electric and magnetic fields in the tightly focused region with *E* = 0.5*E_max_* and *H* = 0.5*H_max_*.

Figure 5 depicts the three-dimensional intensity distribution of the electric and magnetic fields under tight focusing with different polarization orders. The red part represents the electric field, while the blue part represents the magnetic field. From Figure 3, Figure 4 and Figure 5, it is evident that we can obtain the intensity distribution of the electric and magnetic fields by employing the expressions derived above.

**Figure 6:**
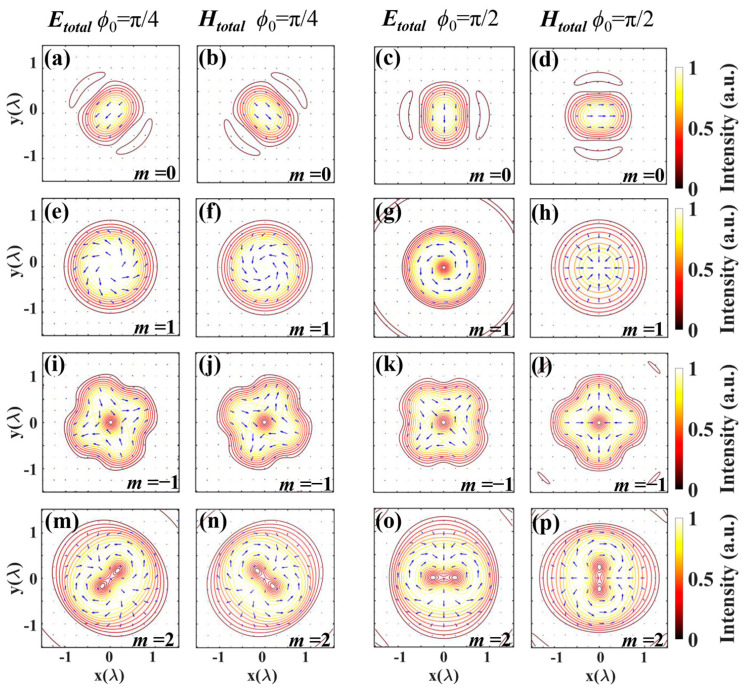
The intensity contour of the electric and magnetic fields in the focal plane for the vector beams: (**a**,**b**) m = 0, *ϕ*_0_ = π/4, (**c**,**d**) m = 0, *ϕ*_0_ = π/2, (**e**,**f**) m = 1, *ϕ*_0_ = π/4, (**g**,**h**) m = 1, *ϕ*_0_ = π/2, (**i**,**j**) m = −1, *ϕ*_0_ = π/4, (**k**,**l**) m = −1, *ϕ*_0_ =π/2, (**m**,**n**) m = 2, *ϕ*_0_ = π/4, (**o**,**p**) m = 2, *ϕ*_0_ = π/2. The short lines indicate the polarization distributions.

Figure 6 illustrates the intensity contour of the electric and magnetic distributions with different polarization orders when the initial phase *ϕ*_0_ is π/4 and π/2, respectively. The arrows represent the polarization distributions. In the top row, where the polarization order *m* = 0 is depicted, the electric field intensity distribution represents linear polarization. When the polarization order *m* = 1 and the initial phase *ϕ*_0_ = π/2, as shown in the right hand of the second row, the electric and magnetic vectors exhibit the azimuthal and radial distributions, respectively. For *m* = 0, −1, and 2, the intensity contour varies with the initial phase change.

### 3.2. Optical Forces on Rayleigh Particle in Tight Focusing of CV Beams

In this section, numerical results are presented to demonstrate the properties of optical forces produced by highly focused partially cylindrical vector (CV) beams exerted on a Rayleigh particle. When a particle is immersed near the focus of light, a compact bright focal spot is expected to capture dielectric particles with a refractive index higher than the ambient index, while a dark core focal spot is expected to capture dielectric particles with a refractive index lower than the ambient index [44]. For numerical calculations, we assume an input power P = 100 mW, a wavelength λ = 840 nm, and an objective lens with a numerical aperture *NA* = 0.9. The captured gold nanoparticles are assumed to have a radius of 30 nm. The relative permittivity of gold (Au) at a wavelength of 840 nm is ε = −25.096 + 2.045i [45].

**Figure 7:**
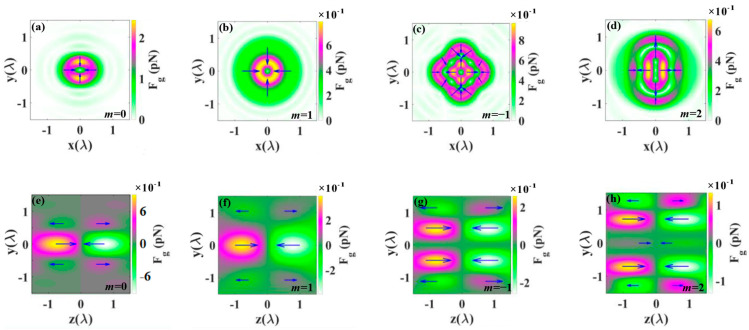
The gradient force distributions produced by highly focused CV beams with *m* equal to 0, 1, −1, and 2. (**a**–**d**) represent the transverse gradient force distributions. (**e**–**h**) represent the longitudinal gradient force distributions. Arrows denote the direction and magnitude of the gradient force.

Figure 7 illustrates the gradient force distributions by tightly focused CV beams in the transverse and longitudinal planes, respectively. Here, the forces on the particles are linear [46]. The transverse force in the figure represents the vector superposition of the force in the x and y directions, defined as (F_x_^2^ + F_y_^2^)^1/2^. On the optical axis, due to the symmetry of light, the gradient force is axially symmetric in the focal plane, and from the color bar of the different images in Figure 7, it is observed that the maximum transverse gradient force decreases with the increase in *m* [47]. Since the intensity region of the electric field enlarges with the increase in *m*, the average amplitude decreases with the increase in *m* at a given input power. In Figure 7, arrows denote the direction and magnitude of the transverse gradient force and longitudinal gradient force. The gradient force results in force balance, allowing the Rayleigh particle to be bound.

**Figure 8:**
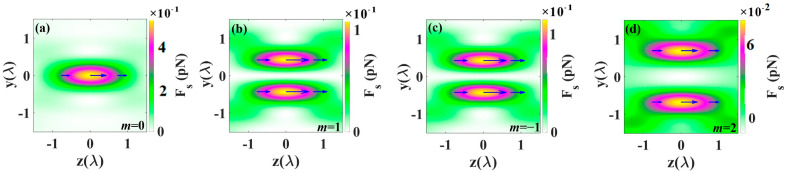
The longitudinal scattering force in the focal plane for different orders *m* of CV beam: (**a**) *m* = 0, (**b**) *m* = 1, (**c**) *m* = −1, (**d**) *m* = 2. Arrows denote the direction and magnitude of the scattering force.

Figure 8 illustrates the longitudinal scattering force in the focal plane for different orders *m* of cylindrical vector (CV) beams. Clearly, the scattering force is primarily generated by the energy flow along the propagation direction. In this study, we do not consider the transverse components of the scattering force in the focal plane. Particularly, it is observed that when *m* = 1, the annular illumination with radial polarization provides a higher gradient force as well as lower scattering forces. Along the optical axis, the scattering force is nearly zero, eliminating a major potential cause of trap destabilization. Furthermore, when *m* = 2, a force opposite to the light propagation direction is formed near the optical axis at the focus position, causing the light not to push the particles towards the light propagation direction. In this case, the effect of light on the particles resembles that of ‘tractor beams’ [48]. The use of light fields with distinct intensity minima where particles can be retained offers an elegant solution. Essentially, any doughnut beam with a phase or polarization singularity and zero light intensity along its axis satisfies the above criterion, facilitating the formation of a stronger pulling force.

**Figure 9:**
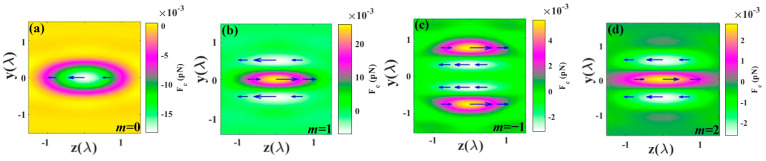
The longitudinal curl-spin force for different orders *m* of CV beam: (**a**) *m* = 0, (**b**) *m* = 1, (**c**) *m* = −1, (**d**) *m* = 2. Arrows denote the direction and magnitude of the curl-spin force.

Figure 9 depicts the longitudinal curl-spin force for different orders *m* of cylindrical vector beams (CVBs). The curl-spin force arises from the gradient in the spatial distribution of beam polarization, resulting from the non-uniform spatial distribution of spin density in the focal region. When the incident light is linearly polarized light, the curl-spin force is zero [49]. However, in a tightly focused system, the curl-spin force is influenced by the polarization of the incident light. As shown in Figure 9, when the polarization of the CVBs changes, the direction of the curl-spin force also changes accordingly. Specifically, when *m* = −1, 1, 2, the direction of the curl-spin force is opposite to that without polarization, both producing forces toward the center of the light field.

It is evident from Figure 7, Figure 8 and Figure 9 that the transverse gradient force significantly outweighs other forces. Consequently, the positions and number of optical trapping sites can be determined by the transverse gradient force. The gradient force is influenced by the intensity distribution, indicating that by altering *m* and thus modifying the distribution of the light field, multiple trapping sites could be achieved.

## 4. Conclusions

In conclusion, we have explored the generation, focusing, and optical force of CV beams. Utilizing the Richards–Wolf vector diffraction integral theory, we derived the integral expressions for the electric field intensity and the magnetic field intensity. Through numerical simulations, we investigated the intensity distribution of the light field and the magnetic field for different orders, providing insights into the 3D intensity distribution under tight focusing. Our findings reveal that by adjusting the order, the spatial distribution and intensity of radial and azimuthal polarization components can be altered, leading to the generation of diverse distributions of electric field components. Furthermore, based on the distribution of the light field and the magnetic field, we characterized the gradient force and explored the scattering force, considering the effects of polarization. We observed that vector beams with arbitrary polarization distributions can generate stable optical trap force potentials, and polarization can influence the direction and magnitude of the force. The non-uniform spatial polarization state of high-order vector beams holds a significant application potential in various fields such as optical control, micro-machining, remote sensing, and space optical communication.

## Data Availability

All data that support the findings of this study are included within the article.
